# Retinal Thickness and Vascular Density Changes in Amyotrophic Lateral Sclerosis Assessed by Optical Coherence Tomography Angiography

**DOI:** 10.3390/biomedicines14071612

**Published:** 2026-07-17

**Authors:** Abdelilah Assialioui, Mónica Povedano, Marta Senau, Isidro Ferrer, Luis Arias

**Affiliations:** 1 Facultat de Medicina i Cienciès de la Salut, Universitat de Barcelona, 08007 Barcelona, Barcelona, Spain; 2 Amyotrophic Lateral Sclerosis Unit, Hospital Universitari de Bellvitge, 08907 L’Hospitalet del Llobregat, Barcelona, Spain; 3 Neurology Department, Consorci Sanitari Alt Penedès-Garraf, 08720 Vilafranca del Penedès, Barcelona, Spain; 4 Ophthalmology Department, Hospital Universitari de Bellvitge, 08720 L’Hospitalet del Llobregat, Barcelona, Spain

**Keywords:** retinal imaging, neurovascular dysfunction, optical coherence tomography angiography, amyotrophic lateral sclerosis, retinal vascular density

## Abstract

**Background:** Amyotrophic lateral sclerosis (ALS) is increasingly recognized as a multisystem disorder involving neurovascular dysfunction. The retina allows in vivo assessment of neurovascular changes. This study evaluated retinal structural and microvascular alterations in ALS using optical coherence tomography (OCT) and optical coherence tomography angiography (OCT-A). **Methods:** This cross-sectional study included 46 participants with ALS and 19 healthy controls. Retinal thickness and vascular density in the superficial and deep retinal capillary plexuses and the choriocapillaris were quantified using OCT and OCT-A. Group comparisons and logistic regression analyses were performed to assess associations with ALS. Subgroup analyses were conducted according to clinical phenotype. **Results:** In total, 124 eyes were analyzed. ALS was associated with increased average retinal thickness (*p* = 0.023) and reduced vascular density in the superficial retinal capillary plexus (*p* = 0.005), deep retinal capillary plexus (*p* < 0.001), and choriocapillaris (*p* = 0.004). In logistic regression analyses, retinal thickness was positively associated with ALS status (OR = 1.42, *p* = 0.023), whereas higher vascular density in the superficial plexus, deep plexus, and choriocapillaris was associated with lower odds of ALS. No significant differences were observed between bulbar- and spinal-onset ALS phenotypes. **Conclusions:** ALS is associated with structural and microvascular retinal alterations detectable by OCT and OCT-A. These findings support the presence of systemic neurovascular dysfunction and highlight retinal imaging as a promising, non-invasive approach for investigating disease mechanisms and developing potential biomarkers.

## 1. Introduction

Amyotrophic lateral sclerosis (ALS) is a progressive and fatal neurodegenerative disorder. It is characterized by degeneration of upper and lower motor neurons, leading to muscle weakness, paralysis, and respiratory failure [1]. Traditionally considered a disease confined to the motor system, ALS is now increasingly recognized as a multisystem disorder involving not only motor neurons but also extra-motor neural networks, glial dysfunction, neuroinflammation, and vascular alterations [2]. Understanding these expanded mechanisms is essential for identifying biomarkers and developing new therapies.

The etiology of ALS is heterogeneous. Approximately 5–10% of cases are familial and associated with pathogenic variants in genes such as *C9ORF72*, *SOD1*, *TARDBP*, and *FUS*, whereas the majority are sporadic [2]. At the cellular level, ALS is characterized by several pathological processes, including abnormal aggregation of TAR DNA-binding protein 43 (TDP-43), mitochondrial dysfunction, oxidative stress, excitotoxicity, and neuroinflammation, all of which contribute to neuronal degeneration [3,4].

Beyond neuronal pathology, evidence supports vascular dysfunction and blood–brain barrier impairment in ALS. Histopathological and experimental studies show endothelial abnormalities, pericyte loss, thickened basement membranes, and increased vascular permeability. These findings have been reported in the spinal cord and brain of individuals with ALS and in animal models [5,6,7]. These vascular changes may appear early and contribute to neuronal injury by impairing perfusion, inducing hypoxia, and disrupting neurovascular homeostasis [8,9].

Microvascular abnormalities in ALS may also extend beyond the central nervous system. Peripheral vascular alterations, including abnormal capillary morphology and density, have been reported in individuals with ALS, supporting the hypothesis of systemic vascular involvement [10]. Histopathological studies have similarly demonstrated structural abnormalities in small vessels, including basement membrane thickening and reduced capillary density, suggesting widespread microvascular pathology [11,12].

The retina provides an accessible window for studying neurovascular structure and function in vivo. Retinal and cerebral microvasculature share similar anatomical and physiological characteristics, and retinal imaging has been widely used as a surrogate marker of cerebral microvascular health in neurological diseases [13]. Optical coherence tomography (OCT) enables high-resolution, non-invasive assessment of retinal structure, whereas OCT angiography (OCT-A) allows quantitative evaluation of retinal microvascular networks without the need for contrast agents [14,15]. Retinal structural alterations have been reported in ALS using OCT, including vascular and retinal layer changes, suggesting involvement of retinal neurovascular pathways [16,17]. However, retinal microvascular changes in ALS remain poorly characterized, and studies using OCT-A in this population are limited [18].

Identification of retinal structural and vascular alterations in ALS may provide insight into disease mechanisms and facilitate the development of non-invasive biomarkers. The aim of this study was to evaluate retinal thickness and vascular density using OCT and OCT-A in participants with ALS and healthy controls. Additionally, the study investigated whether retinal changes differed according to clinical phenotype, specifically bulbar-onset versus spinal-onset ALS. It was hypothesized that ALS would be associated with reduced retinal vascular density and structural alterations, reflecting disease-related neurovascular involvement.

## 2. Materials and Methods

### 2.1. Study Design

This cross-sectional observational study evaluated structural and vascular retinal changes in participants with ALS using OCT and OCT-A. Participants with ALS were compared with healthy controls. Subgroup analyses were performed according to disease phenotype (bulbar-onset versus spinal-onset ALS).

### 2.2. Participants with ALS

Adults with ALS aged 18 years or older were recruited from the multidisciplinary motor neuron disease unit at Bellvitge University Hospital. All met the revised El Escorial criteria for probable or definite ALS [19,20]. Participants with other motor neuron disorders were excluded.

All participants with ALS underwent a standardized clinical evaluation. This included neurological examination, cranial and cervical MRI, electrophysiological assessment with electroneurography and electromyography, blood tests, and lumbar puncture. Transcranial magnetic stimulation was performed when clinically indicated. Diagnosis was confirmed based on clinical findings, electrophysiological results, and longitudinal follow-up.

Participants were classified by site of symptom onset: spinal, bulbar, or respiratory. Spinal-onset ALS was defined by initial limb weakness with clinical signs including muscle atrophy, weakness, fasciculations, and hyperreflexia. Bulbar-onset ALS was defined by dysarthria, dysphagia, or tongue fasciculations at presentation. Respiratory-onset ALS was defined by dyspnea or orthopnea at presentation, with or without limb or bulbar involvement. Clinical and imaging assessments were performed during routine clinical visits.

### 2.3. Healthy Controls

Healthy controls were recruited from employees of Bellvitge University Hospital and were selected to achieve an age distribution similar to that of the ALS cohort. Controls had no history of neurological or ocular disease. They also had no systemic conditions known to affect OCT or OCT-A results.

### 2.4. Exclusion Criteria

Participants were excluded if they did not give written consent; had arterial hypertension or diabetes mellitus; had ocular diseases such as glaucoma, retinal detachment, or macular degeneration; had retinal or systemic conditions affecting microvasculature or thickness; could not maintain head position during imaging; or had early-stage disease, according to predefined clinical criteria.

### 2.5. Clinical and Demographic Data

All participants underwent a structured clinical interview to collect demographic and clinical information, including participant identification code, sex, date of birth, smoking status (current or former), and medical history of arterial hypertension, hyperlipidemia, diabetes mellitus, and ocular diseases. For participants with ALS, additional disease-specific data were recorded, including age at symptom onset, disease duration (months since symptom onset), and site of onset (bulbar, spinal, or respiratory).

### 2.6. Optical Coherence Tomography (OCT)

OCT imaging was performed using a swept-source OCT (SS-OCT) system (DRI OCT Triton, Topcon Healthcare, Tokyo, Japan), which uses a 1050-nm wavelength swept laser source. Macular scans were centered on the fovea. Automated segmentation algorithms were used to quantify central foveal thickness (μm) and retinal average thickness (μm). All images were reviewed by an experienced ophthalmologist masked to participant group allocation to ensure image quality and segmentation accuracy.

### 2.7. Optical Coherence Tomography Angiography (OCT-A)

Retinal microvascular parameters were assessed using the OCT-A module of the same SS-OCT system (DRI OCT Triton, Topcon Healthcare, Tokyo, Japan). A 6 × 6 mm macular scan protocol centered on the fovea was used. Vascular density was evaluated in the superficial and deep retinal capillary plexuses, outer retina, and choriocapillaris, as well as foveal vascular density in the superficial and deep plexuses. Scans with poor signal quality or motion artifacts were excluded.

**Figure 1:**
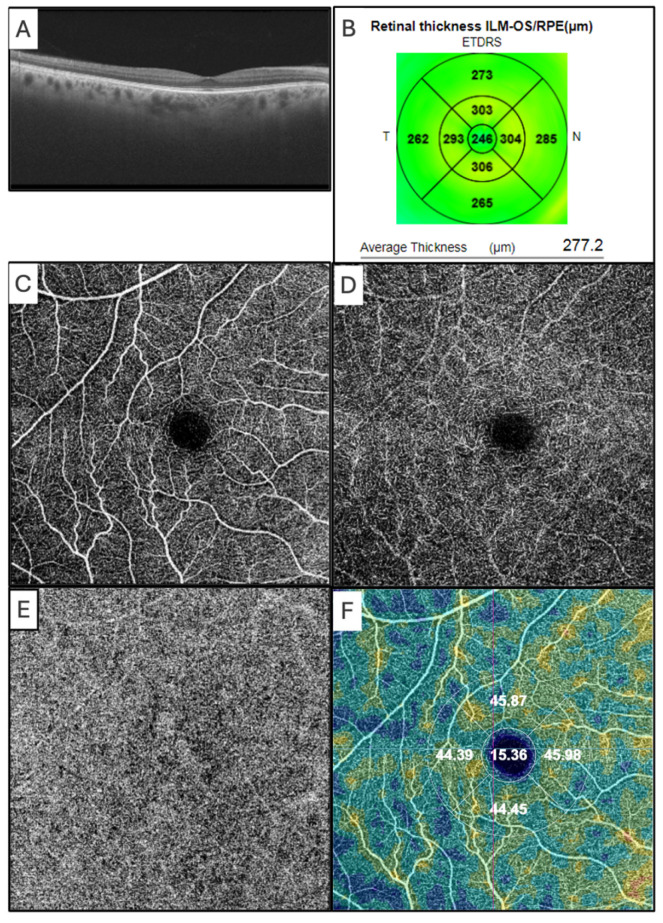
Representative OCT and OCT-A images used for retinal thickness and vascular density assessment. Representative de-identified images from one participant are shown, including (**A**) structural OCT B-scan centered on the fovea, (**B**) retinal thickness map with Early Treatment Diabetic Retinopathy Study (ETDRS) sectors, (**C**) superficial vascular plexus, (**D**) deep vascular plexus, (**E**) choriocapillaris, and (**F**) OCT-A vascular density map. These images illustrate the acquisition and segmentation outputs used for the quantitative analysis of retinal thickness and vascular density.

Foveal vascular density was automatically quantified using the device’s built-in software (IMAGEnet 6, version 1.16E; Topcon Healthcare). Vascular density in the superficial and deep retinal capillary plexuses, outer retina, and choriocapillaris was quantified using ImageJ software (version 1.53; National Institutes of Health, Bethesda, MD, USA). Representative OCT and OCT-A images used for retinal thickness and vascular density assessment are shown in Figure 1.

### 2.8. Quantification of Vascular Density Using Gray-Scale Analysis

Vascular density was quantified from en face OCT-A images using ImageJ software (National Institutes of Health, Bethesda, MD, USA; https://imagej.net, accessed on 21 July 2021). En face images of the superficial and deep retinal capillary plexuses, outer retina, and choriocapillaris were exported from the OCT-A device and analyzed offline. A region of interest centered on the fovea corresponding to the 6 × 6 mm scan area was defined using the polygon selection tool and recorded in the ROI Manager to ensure consistency across images. The mean gray value within each region of interest, representing the average flow signal intensity, was measured using the Analyze > Measure function. All images were analyzed using identical parameters to ensure reproducibility across participants and groups. Images with motion artifacts were excluded, and motion correction algorithms were not applied to avoid introducing processing-related variability.

### 2.9. Statistical Analysis

For categorical variables, frequencies and percentages were calculated, together with exact 95% confidence intervals. For continuous variables, the mean and standard deviation (SD), 95% confidence interval (CI) for the mean, and median and interquartile range (IQR) were calculated.

Group comparisons were performed according to variable type. For continuous variables, the Mann–Whitney U test was used. For categorical variables, a chi-square test or Fisher’s exact test was applied when expected cell counts were below 5.

The following variables were divided by 10 to facilitate interpretation in the models: central foveal thickness (µm), retinal average thickness (µm), superficial plexus vascular density, deep plexus vascular density, outer retinal vascular density, choriocapillaris vascular density, superficial plexus foveal vascular density, and deep plexus foveal vascular density. Thus, a 1-unit increase in these scaled variables corresponds to a 10-unit increase on the original scale, allowing for a more intuitive interpretation of the estimated coefficients.

OCT and OCT-A measurements were analyzed at the eye level. Both eyes were included when image quality met the predefined criteria. Structural OCT and OCT-A images were assessed independently. Because measurements from fellow eyes are not statistically independent, the logistic regression models were estimated using cluster-robust standard errors to account for the within-subject correlation between eyes.

All analyses were performed using R software (R version 4.5.1 (2025-06-13 ucrt), Copyright (C) 2025 The R Foundation for Statistical Computing).

## 3. Results

### 3.1. Baseline Characteristics

A total of 65 participants were included, with 19 controls (29.2%) and 46 individuals with ALS (70.8%). Regarding phenotype, ALS cases were distributed between bulbar onset (28.3%) and spinal onset (71.7%).

Sex distribution was similar across groups, with women representing 63.2% of the control group and 47.8% of the ALS group (*p* = 0.264). Consistent with the study design, there was no significant difference in mean age between healthy controls (58.1 ± 9.5 years) and participants with ALS (59.1 ± 10.0 years; *p* = 0.639).

Smoking habits were also comparable between groups. Most participants were nonsmokers (84.4%), with no significant differences in the proportion of current smokers (21.1% in controls vs. 13.3% in ALS; *p* = 0.466) or former smokers (26.3% vs. 26.7%; *p* = 0.977) (Table 1).

### 3.2. OCT and OCT-A Analysis

OCT and OCT-A examinations were attempted in both eyes of all participants. Overall, 124 eyes (88 ALS eyes and 36 control eyes) met the quality criteria for at least one imaging analysis. Structural OCT and OCT-A were analyzed independently; therefore, the number of eyes included differed according to the quality criteria for each imaging modality. Retinal thickness measurements were available for 115 eyes, whereas the number of eyes included in each OCT-A analysis varied according to scan quality and the retinal vascular layer evaluated (Table 2). ALS participants exhibited higher central foveal thickness than controls, although the difference did not reach statistical significance (25.7 ± 2.4 vs. 24.8 ± 1.6 μm; *p* = 0.074). Retinal average thickness was significantly greater in the ALS group than in controls (28.0 ± 1.5 vs. 27.3 ± 1.3 μm; *p* = 0.023).

The superficial plexus vascular density was significantly lower in the ALS group than in healthy controls (9.1 ± 0.5 vs. 9.5 ± 0.5; *p* = 0.005). The deep plexus vascular density was also lower in the ALS group than in healthy controls (8.7 ± 0.5 vs. 9.2 ± 0.7; *p* < 0.001). The choriocapillaris vascular density was significantly lower in the ALS group than in healthy controls (11.0 ± 0.4 vs. 11.3 ± 0.2; *p* = 0.004).

**Figure 2:**
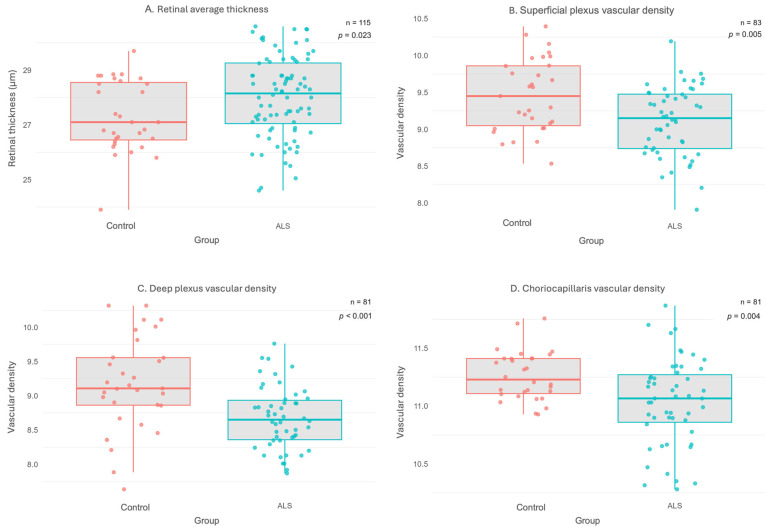
Comparison of retinal vascular density and retinal average thickness between healthy controls and participants with ALS. Boxplots show (**A**) retinal average thickness, (**B**) superficial plexus vascular density, (**C**) deep plexus vascular density, and (**D**) choriocapillaris vascular density. Participants with ALS exhibited significantly lower vascular density in the superficial and deep retinal capillary plexuses and the choriocapillaris, as well as significantly higher retinal average thickness, compared with healthy controls. The number of observations included in each analysis (*n*) and the corresponding *p*-value are shown in each panel. Central lines indicate medians; boxes represent interquartile ranges (IQRs); whiskers denote 1.5 × IQR; and dots represent individual analyzed eyes.

No statistically significant differences were observed in outer retinal vascular density (3.6 ± 0.2 vs. 3.5 ± 0.2; *p* = 0.226), superficial plexus foveal vascular density (2.0 ± 0.3 vs. 1.9 ± 0.5; *p* = 0.122), or deep plexus foveal vascular density (1.7 ± 0.4 vs. 1.8 ± 0.5; *p* = 0.677). These findings are summarized in Table 2 and Figure 2.

### 3.3. Univariable Logistic Regression Analyses

Separate univariable binary logistic regression models were fitted to examine the association between retinal and demographic parameters and ALS status. Retinal average thickness was positively associated with ALS status (OR = 1.42, 95% CI: 1.06–1.94; *p* = 0.023), indicating that greater retinal thickness was associated with higher odds of ALS. In contrast, higher vascular density in the superficial retinal capillary plexus (OR = 0.21, 95% CI: 0.07–0.56; *p* = 0.003), deep retinal capillary plexus (OR = 0.20, 95% CI: 0.07–0.48; *p* = 0.001), and choriocapillaris (OR = 0.10, 95% CI: 0.02–0.47; *p* = 0.007) was associated with lower odds of ALS. Neither age (OR = 1.00, 95% CI: 0.96–1.04; *p* = 0.91) nor male sex (OR = 2.19, 95% CI: 0.99–5.05; *p* = 0.058) was significantly associated with ALS status, although male sex showed a non-significant trend toward higher odds of ALS. These findings suggest that increased retinal thickness and reduced vascular density in the superficial and deep retinal capillary plexuses and the choriocapillaris are associated with ALS status (Table 3).

### 3.4. Phenotype Analysis

A significant difference in sex distribution was observed between the bulbar- and spinal-onset groups (*p* = 0.029), with a higher proportion of women in the bulbar group (66.7%) than in the spinal group (40.6%). Age differed significantly between groups. Participants with bulbar-onset ALS were older than those with spinal-onset ALS (62.2 ± 6.6 vs. 57.5 ± 10.7 years; *p* = 0.018). Age at onset was higher in the bulbar-onset group than in the spinal-onset group (60.0 ± 6.9 vs. 53.8 ± 13.1 years; *p* = 0.026). No significant differences were found in disease duration (*p* = 0.491) or smoking status (*p* > 0.05).

**Figure 3:**
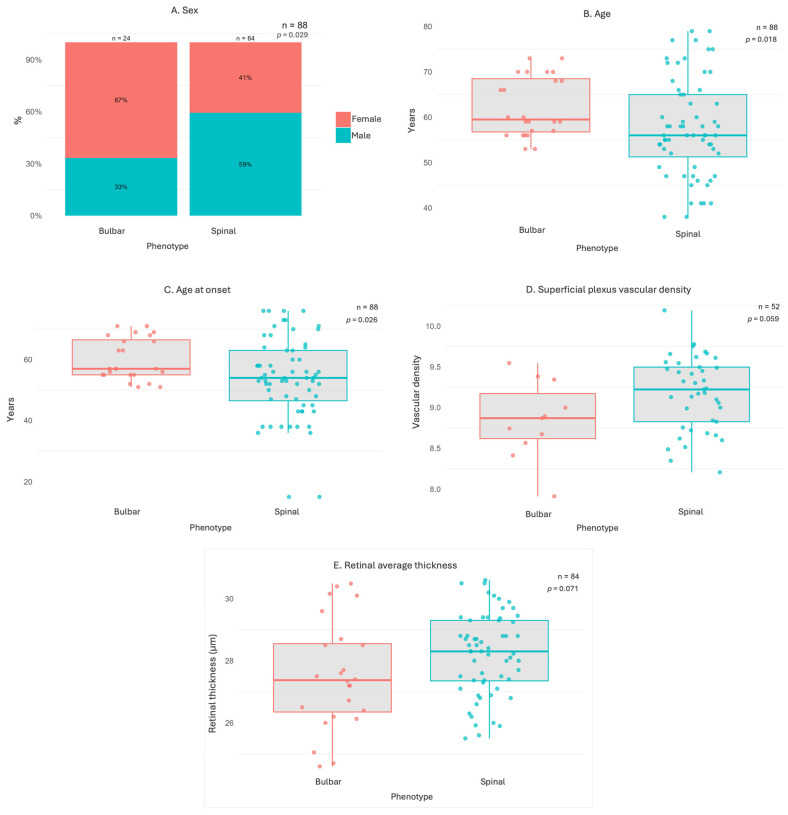
Comparison of demographic, clinical, OCT, and OCT-A characteristics between participants with bulbar- and spinal-onset ALS. (**A**) Sex distribution, (**B**) age, (**C**) age at onset, (**D**) superficial plexus vascular density, and (**E**) retinal average thickness. Participants with bulbar-onset ALS were significantly older and had a higher age at onset than those with spinal-onset ALS. Retinal average thickness and superficial plexus vascular density tended to be lower in the bulbar-onset group, although these differences did not reach statistical significance. The number of observations included in each analysis (*n*) and the corresponding *p*-value are shown in each panel. In panel (**A**), stacked bars represent the percentage of female and male participants. In panels (**B**–**E**), central lines indicate medians; boxes represent interquartile ranges (IQRs); whiskers extend to 1.5 × IQR; and dots represent individual observations.

Regarding retinal parameters, none of the comparisons between bulbar- and spinal-onset phenotypes reached statistical significance, although non-significant trends were observed. Retinal average thickness tended to be greater in spinal-onset ALS than in bulbar-onset ALS (28.2 ± 1.3 vs. 27.5 ± 1.7 µm; *p* = 0.071). Superficial plexus vascular density tended to be lower in the bulbar-onset group than in the spinal-onset group (8.8 ± 0.5 vs. 9.2 ± 0.4; *p* = 0.059). Superficial plexus foveal vascular density showed a non-significant trend toward lower values in the bulbar-onset group compared with the spinal-onset group (1.9 ± 0.4 vs. 2.1 ± 0.2; *p* = 0.072). Other OCT and OCT-A parameters did not differ significantly between phenotypes (*p* > 0.05). These findings are summarized in Table 4 and Figure 3.

## 4. Discussion

Our findings show concurrent structural and microvascular retinal changes in the ALS cohort. Using OCT and OCT-A, we identified a significant increase in average retinal thickness, accompanied by a significant reduction in vascular density within the superficial and deep retinal capillary plexuses and the choriocapillaris compared with healthy controls. OCT and OCT-A may therefore represent valuable tools for detecting and monitoring subclinical neurovascular alterations, improving understanding of disease mechanisms, and potentially contributing to the evaluation of future therapeutic strategies.

Vascular impairment in ALS has been extensively described within the central nervous system. Histopathological and experimental investigations have documented endothelial abnormalities, pericyte degeneration, basement membrane alterations, and disruption of the blood–brain and blood–spinal cord barriers in individuals with ALS and in transgenic animal models [5,6,7,12,21]. Recently, capillary abnormalities have been detected in individuals with ALS using nailfold videocapillaroscopy [10].

From a mechanistic perspective, microvascular rarefaction may reflect impaired angiogenic maintenance and progressive capillary regression, consistent with barrier disruption described in individuals with ALS and experimental models [12,21]. Recent experimental evidence further links TDP-43 biology to endothelial stability, demonstrating that TDP-43 is required for sprouting angiogenesis, vascular barrier integrity, and blood vessel stability. Specifically, deletion of TDP-43 in postnatal endothelial cells leads to retinal hypovascularization due to defects in vessel sprouting, which are associated with reduced endothelial cell proliferation and migration. In mature blood vessels, loss of TDP-43 disrupts the blood–brain barrier and triggers vascular degeneration. These vascular defects are accompanied by an inflammatory response in the central nervous system (CNS), characterized by microglial and astrocyte activation [22]. In turn, blood–CNS barrier breakdown promotes vascular leakage, interstitial fluid accumulation, and inflammatory remodeling [23], processes that may lead to reactive gliosis and structural retinal thickening despite vascular loss.

These findings provide in vivo evidence that ALS extends beyond motor neuron degeneration and involves systemic neurovascular impairment. The concordance between peripheral microvascular alterations and the retinal changes observed in our OCT-A analysis supports the concept of a generalized vascular process rather than one confined to the central nervous system. Together, these observations are consistent with a model of neurovascular uncoupling in ALS, in which endothelial dysfunction and microvascular compromise contribute to secondary inflammatory and structural alterations. In this context, retinal structural and vascular measures may represent clinically relevant, non-invasive biomarkers of ALS-related neurovascular dysfunction.

Structural retinal alterations in ALS have been reported in several OCT studies, although results have been heterogeneous. Early investigations identified subtle reductions in macular thickness and the retinal nerve fiber layer (RNFL), as well as a marked thinning of the inner nuclear layer (INL) [24]. Subsequent studies demonstrated that the macular RNFL was significantly thinner in participants with ALS compared with healthy controls and correlated with pulmonary function test results [25]. Additional work has reported reductions in RNFL thickness that differed between the right and left eyes of individuals with ALS, suggesting that asymmetric central nervous system involvement in ALS is not confined to the motor system [26]. Another study identified a reduction in RNFL thickness in individuals with ALS; however, this finding did not correlate with Amyotrophic Lateral Sclerosis Functional Rating Scale–Revised (ALSFRS-R) score, ALSFRS-R progression rate, forced vital capacity, intraocular pressure, or visual acuity [27]. Multimodal imaging studies combining OCT and diffusion tensor imaging have further revealed a significant thinning of the INL and the RNFL [28]. In addition to structural changes, alterations in retinal vascular morphology have also been described, including thickening of retinal vessel walls, as assessed using Spectralis-OCT images [16].

When comparing ALS phenotypes, we found no statistically significant differences between bulbar- and spinal-onset subgroups. However, trends toward lower vascular density and reduced retinal thickness were observed in bulbar-onset ALS. Given that bulbar-onset ALS is often associated with faster progression and poorer prognosis [29], these tendencies may indicate phenotype-specific neurovascular vulnerability, although larger cohorts, longitudinal designs, and studies integrating retinal imaging with molecular biomarkers such as TDP-43 are needed to confirm these findings and further clarify the underlying pathophysiological mechanisms.

Methodological differences may partly explain discrepancies between our findings and previous OCT-A studies. Our cohort consisted of individuals with ALS at intermediate to advanced disease stages who were able to maintain adequate head positioning during image acquisition. Consequently, a substantial proportion of potential participants were excluded due to poor image quality related to motion artifacts secondary to cervical weakness. We also restricted inclusion to participants fulfilling the revised El Escorial criteria for probable or definite ALS, resulting in a clinically well-defined cohort. These design characteristics may have enhanced the detection of microvascular alterations. In contrast, Cennamo et al. reported no significant differences in macular or peripapillary vascular density between ALS and controls, which may reflect differences in cohort composition and study design. Notably, their inclusion of individuals meeting “probable laboratory-supported” diagnostic criteria may have introduced greater clinical heterogeneity [18].

Taken together, evidence from neuropathology, peripheral vascular studies, and retinal imaging supports a model of ALS involving systemic neurovascular dysfunction. In this context, OCT-A-derived retinal vascular measures may provide a non-invasive window into disease-related microvascular alterations and represent a promising candidate biomarker.

## 5. Conclusions

Our findings demonstrate that ALS is associated with concurrent structural and microvascular retinal alterations detectable by OCT and OCT-A, including increased retinal thickness and reduced vascular density in the superficial and deep retinal capillary plexuses and the choriocapillaris. These results support the concept of ALS as a disorder involving systemic neurovascular dysfunction. Retinal imaging may provide a non-invasive approach to investigate disease-related vascular changes and may represent a promising candidate biomarker.

## Figures and Tables

**Table 1:** Demographic and clinical characteristics of healthy controls and participants with ALS.

Variable	Healthy Controls (*n* = 19)	ALS (*n* = 46)	*p*-Value
Phenotype			
Bulbar	–	13 (28.3%) CI: 16.0–43.5	
Spinal	–	33 (71.7%) CI: 56.5–84.0	
Sex			0.264
Female	12 (63.2%) CI: 38.4–83.7	22 (47.8%) CI: 32.9–63.1	
Male	7 (36.8%) CI: 16.3–61.6	24 (52.2%) CI: 36.9–67.1	
Age (years)	58.1 (9.5) CI: 53.5–62.6 57.0 [49.5, 64.0]	59.1 (10.1) CI: 56.1–62.1 58.0 [54.0, 67.5]	0.639
Age at onset (years)	–	55.9 (12.1) CI: 52.3–59.5	
Disease duration (months)	–	28.5 (16.2) CI: 23.7–33.4	
Current smoker			0.466
No	15 (78.9%) CI: 54.4–93.9	39 (86.7%) CI: 73.2–94.9	
Yes	4 (21.1%) CI: 6.1–45.6	6 (13.3%) CI: 5.1–26.8	
Former smoker			0.977
No	14 (73.7%) CI: 48.8–90.9	33 (73.3%) CI: 58.1–85.4	
Yes	5 (26.3%) CI: 9.1–51.2	12 (26.7%) CI: 14.6–41.9	

Values are presented as *n* (%) with exact 95% confidence intervals (CIs) for categorical variables and as mean ± standard deviation (SD), 95% confidence interval (CI), and median [interquartile range (IQR)] for continuous variables. Categorical variables were compared using Fisher’s exact test or Pearson’s chi-square test, as appropriate. Continuous variables were compared using the Mann–Whitney U test. Variables reported only for the ALS group were presented descriptively and were not statistically compared with healthy controls.

**Table 2:** Comparison of OCT and OCT-A parameters between healthy controls and participants with ALS.

Variable	Healthy Controls (*n* = 36 Eyes)	ALS (*n* = 88 Eyes)	*p*-Value	*n*
Central foveal thickness (μm)	24.8 (1.6) CI: 24.2–25.4 24.8 [23.6, 25.9]	25.7 (2.4) CI: 25.2–26.2 25.6 [23.9, 27.3]	0.074	115
Retinal average thickness (μm)	27.3 (1.3) CI: 26.8–27.8 27.1 [26.4, 28.6]	28.0 (1.5) CI: 27.7–28.3 28.1 [27.0, 29.3]	0.023	115
Superficial plexus vascular density	9.5 (0.5) CI: 9.3–9.6 9.5 [9.0, 9.9]	9.1 (0.5) CI: 9.0–9.2 9.2 [8.7, 9.5]	0.005	83
Deep plexus vascular density	9.2 (0.7) CI: 8.9–9.4 9.1 [8.9, 9.6]	8.7 (0.5) CI: 8.5–8.8 8.7 [8.4, 8.9]	<0.001	81
Outer retinal vascular density	3.5 (0.2) CI: 3.5–3.6 3.5 [3.4, 3.7]	3.6 (0.2) CI: 3.5–3.6 3.6 [3.5, 3.7]	0.226	83
Choriocapillaris vascular density	11.3 (0.2) CI: 11.2–11.3 11.2 [11.1, 11.4]	11.0 (0.4) CI: 10.9–11.1 11.1 [10.9, 11.3]	0.004	81
Superficial plexus foveal vascular density	1.9 (0.5) CI: 1.7–2.1 1.9 [1.8, 2.1]	2.0 (0.3) CI: 2.0–2.1 2.0 [1.9, 2.2]	0.122	81
Deep plexus foveal vascular density	1.8 (0.5) CI: 1.6–1.9 1.9 [1.3, 2.1]	1.7 (0.4) CI: 1.6–1.9 1.7 [1.4, 1.9]	0.677	75

Values are presented as mean ± standard deviation (SD), 95% confidence interval (CI) for the mean, and median [interquartile range (IQR)]. The final *n* column indicates the number of eyes included in each analysis. Continuous variables were compared using the Mann–Whitney U test.

**Table 3:** Univariable logistic regression analyses of retinal and demographic parameters associated with ALS status.

Variable	OR	95% CI	*p*-Value	*n*
Retinal average thickness (µm)	1.42	1.06–1.94	0.023	115
Superficial plexus vascular density	0.21	0.07–0.56	0.003	83
Deep plexus vascular density	0.20	0.07–0.48	0.001	81
Choriocapillaris vascular density	0.10	0.02–0.47	0.007	81
Age (years)	1.00	0.96–1.04	0.91	124
Male sex	2.19	0.99–5.05	0.058	124

Abbreviations: OR, odds ratio; CI, confidence interval. Odds ratios, confidence intervals, and *p*-values were obtained from separate univariable binary logistic regression models, with healthy controls as the reference group. OCT and OCT-A variables were divided by 10 before model fitting; therefore, ORs represent the change in odds associated with a 10-unit increase in the original measurement scale. For sex, the OR refers to male sex compared with female sex.

**Table 4:** Comparison of demographic, clinical, OCT, and OCT-A characteristics between bulbar- and spinal-onset ALS.

Variable	Bulbar	Spinal	*p*-Value	*n*
Sex			0.0294	88
Female	16 (66.7%) CI: 44.7–84.4	26 (40.6%) CI: 28.5–53.6		
Male	8 (33.3%) CI: 15.6–55.3	38 (59.4%) CI: 46.4–71.5		
Age	62.2 (6.6) CI: 59.5–65.0 59.5 [56.8, 68.5]	57.5 (10.7) CI: 54.8–60.1 56 [51.2, 65]	0.018	88
Age at onset	60.0 (6.9) CI: 57.1–62.9 57 [55, 66.5]	53.8 (13.1) CI: 50.5–57.1 54 [46.5, 63]	0.026	88
Disease duration (months)	27.0 (14.3) CI: 21.0–33.0 23 [16.8, 34.2]	29.7 (17.0) CI: 25.5–34.0 24 [20, 36]	0.491	88
Current smoker			0.723	87
No	21 (91.3%) CI: 72.0–98.9	55 (85.9%) CI: 75.0–93.4		
Yes	2 (8.7%) CI: 1.1–28.0	9 (14.1%) CI: 6.6–25.0		
Former smoker			0.294	87
No	15 (65.2%) CI: 42.7–83.6	49 (76.6%) CI: 64.3–86.2		
Yes	8 (34.8%) CI: 16.4–57.3	15 (23.4%) CI: 13.8–35.7		
Central foveal thickness (µm)	25.2 (1.8) CI: 24.4–25.9 25.5 [24, 26.6]	25.9 (2.6) CI: 25.3–26.6 25.8 [23.8, 27.7]	0.218	84
Retinal average thickness (µm)	27.5 (1.7) CI: 26.8–28.3 27.4 [26.3, 28.6]	28.2 (1.3) CI: 27.9–28.6 28.3 [27.4, 29.3]	0.071	84
Superficial plexus vascular density	8.8 (0.5) CI: 8.5–9.2 8.9 [8.6, 9.2]	9.2 (0.4) CI: 9.0–9.3 9.2 [8.8, 9.5]	0.059	52
Deep plexus vascular density	8.5 (0.5) CI: 8.2–8.9 8.5 [8.2, 8.7]	8.7 (0.4) CI: 8.6–8.9 8.7 [8.4, 9.0]	0.185	50
Outer retinal vascular density	3.6 (0.2) CI: 3.5–3.7 3.6 [3.5, 3.7]	3.6 (0.2) CI: 3.5–3.6 3.6 [3.5, 3.7]	0.877	52
Choriocapillaris vascular density	11.0 (0.4) CI: 10.7–11.2 10.9 [10.8, 11.1]	11.1 (0.4) CI: 10.9–11.2 11.1 [10.9, 11.3]	0.422	51
Superficial plexus foveal vascular density	1.9 (0.4) CI: 1.6–2.2 1.9 [1.6, 2.1]	2.1 (0.2) CI: 2.0–2.2 2.1 [2.0, 2.2]	0.072	51
Deep plexus foveal vascular density	1.6 (0.6) CI: 1.2–2.0 1.5 [1.4, 1.9]	1.8 (0.4) CI: 1.7–1.9 1.8 [1.6, 1.9]	0.436	45

Values are presented as *n* (%) with exact 95% confidence intervals (CIs) for categorical variables and as mean ± standard deviation (SD), 95% confidence interval (CI) for the mean, and median [interquartile range (IQR)] for continuous variables. The final *n* column indicates the number of observations included in each analysis. Categorical variables were compared using Pearson’s chi-square test or Fisher’s exact test, as appropriate. Continuous variables were compared using the Mann–Whitney U test.

## Data Availability

The data presented in this study are available from the corresponding author upon reasonable request. The data are not publicly available due to privacy and ethical restrictions related to participant confidentiality.

## Abbreviations

The following abbreviations are used in this manuscript:

ALS	Amyotrophic lateral sclerosis
OCT	Optical coherence tomography
OCT-A	Optical coherence tomography angiography
SS-OCT	Swept-source optical coherence tomography
ETDRS	Early Treatment Diabetic Retinopathy Study
CI	Confidence interval
IQR	Interquartile range
OR	Odds ratio
RNFL	Retinal nerve fiber layer
INL	Inner nuclear layer
ALSFRS-R	Amyotrophic Lateral Sclerosis Functional Rating Scale–Revised
